# Does Sterilization Affect the Performance of Single-Step Resin Composite Polishers?

**DOI:** 10.3390/dj11050128

**Published:** 2023-05-08

**Authors:** Christina Papadopoulou, Maria Anagnostou, Konstantinos Masouras, Vasileios Margaritis, Charikleia Paximada

**Affiliations:** 1 Independent Researcher, 11527 Athens, Greece; 2 Department of Operative Dentistry, School of Dentistry, National and Kapodistrian University of Athens, 11527 Athens, Greece; 3 SSM Research Center, Swiss School of Management, 6500 Bellinzona, Switzerland

**Keywords:** gloss, polishing, resin-composites, roughness, sterilization

## Abstract

(1) Background: Single-step polishers are used extensively for resin-composite polishing. The purpose of this study was to evaluate the effect of sterilization on their performance. (2) Methods: Optrapol Next Generation/Ivoclar-Vivadent, Jazz Supreme/SS White, Optishine Brush/Kerr and Jiffy Polishing Brush/Ultradent were used for polishing a nanohybrid resin composite (IPS Empress Direct/Ivoclar-Vivadent). Polishers (n = 40) were microscopically inspected before use. After polishing, surface roughness (Sa, Sz, Sdr, Sci) and gloss were determined. Polishers were subsequently sterilized and microscopically re-examined. The process was repeated four times on new samples (n = 200). Data were analyzed using the Friedman test and Wilcoxon post hoc test, at α = 0.05. (3) Results: Optrapol’s performance improved after the first sterilization for Sa and gloss, whereas it declined after the fourth sterilization for Sa. Jazz’s, improved after the second sterilization for Sa and gloss and after the third sterilization for Sdr. An improvement trend was observed for Optishine after the first sterilization, but not statistically significant. Sa, Sz, and gloss declined after the fourth sterilization. Jiffy’s performance was inconsistent, with a trend of performance loss after the fourth sterilization. (4) Conclusions: Performance of all polishing systems improved after the initial sterilization, but deteriorated after the fourth sterilization cycle. However, their performance can be considered clinically acceptable for a longer period of use.

## 1. Introduction

Resin composites are currently the main direct restorative materials for both anterior and posterior restorations. The significant improvement of their mechanical and esthetic properties has led to an almost complete replacement of amalgam [1]. The pursuit of high-quality, long-lasting restorations is essential, as indicated by the development of quality assurance systems for the assessment and improvement of dental care [2]. Finishing and polishing resin composites are very important for the biological, functional, and esthetic success, as well as for the longevity of the restorations. Clinicians have a variety of means for finishing resin composites, such as multi-fluted carbide burs and fine diamond burs. Polishing can be achieved by using discs, rubbers, strips, brushes, wheels, and pastes [3].

Surface roughness and gloss have been extensively used to determine the efficacy of polishing means [4]. Smoother surfaces are less likely to retain dental plaque and pigments, and, therefore, reduce the risk of gum inflammation and tooth caries [5,6,7]. No significant differences in the accumulation of dental plaque have been recorded, when Ra ranged from 0.7 μm to 1.4 μm [8,9]. It has also been reported that when Ra values were below 0.2 μm, the plaque accumulation could not be further reduced. Thus, 0.2 μm is considered a threshold for clinically acceptable restorations [10,11].

Finishing and polishing significantly affect the glossiness of a surface, as they are related to light reflection. Roughness and reflectivity have an inversely proportional relationship, as increased micro-roughness leads to reduced surface reflectivity [12]. Therefore, factors that regulate the polishing ability of resin composites, such as some inherent characteristics related to their composition and the type of the polishing means, are those that mainly determine the reflectivity of their surface.

The presence of a softer polymer matrix and harder fillers, which get ground at a different degree during polishing, complicate the production of smooth surfaces. The faster abrasion of the weaker polymer matrix leads to exposure of unsupported fillers [13]. Thus, the roughness of a resin composite surface is essentially determined by the size of the fillers and by the difference in hardness between the fillers and polymer matrix [12].

Surface roughness is measured using devices such as profilometers [14,15], atomic force microscopes (AFM) [7,16], optical microscopes [17], and scanning electron microscopes (SEM) [16]. Gloss is measured using glossmeters that measure the reflection of the incident light by the surface [14].

Initially, polishing was a 3- or a 2-step procedure, using progressively finer means [18,19,20]. The omission of using finishing means prior to polishing means is not recommended, as it would lead to rougher surfaces and excessive wear of the latter [3].

Lately, novel means, specified as “single-step”, have appeared to simplify and shorten the polishing procedure [21,22,23,24]. Differences exist among them regarding the composition and size of the abrasive grains as well as the matrix in which those grains are embedded or attached on. The polishing outcome may vary depending on the rotation speed and the pressure applied, while some of them are recommended to be used under water spray [25].

Data in the literature are controversial regarding the effectiveness of polishing means. There are studies supporting the use of a sequence of decreasing abrasive means [14,26], while others show that one-step polishing systems are equivalent or sometimes better than certain multi-step systems [7,21,22,23,24,27,28].

The tooth surface morphological variations rendered the fabrication of different shapes of polishing means necessary, such as points, discs, and cups. Furthermore, the fine anatomy of the posterior occlusal surfaces pointed out the need for the evolution of thinner polishing means, which could get in contact with the resin composite restorations even at narrow areas, such as the pits and fissures. So, polishing brushes of different shapes and bristle length were fabricated. 

The intraoral use of the ones proposed for multiple uses makes sterilization necessary in order to avoid the spread of any infection. Antiseptic solutions have not always proven effective in destroying all pathogenic viruses, micro-organisms, and their seeds [29,30]. Therefore, an autoclave sterilization, which has been proven fully effective, has become the standard procedure [31]. However, heat rise and humidity may have an impact on the structure and, subsequently, the polishing ability of both multi-step and single-step systems over time [32].

In general, manufacturers do not propose a maximum number of uses and, consequently, sterilization cycles for polishing means. Few data are available about the impact of sterilization on their polishing ability. The purpose of the present study was to examine the effect of sterilization on the polishing effectiveness of four single-step polishing systems.

The null hypothesis tested was that repeated sterilization cycles and the following use do not downgrade the polishing ability of single-step polishing systems for resin composites.

## 2. Methods

Four single-step polishing systems were used to polish a nanohybrid resin composite (Table 1).

Two hundred disc-specimens of 5 mm diameter and 3 mm thickness were fabricated. The specimens were photopolymerized for 40 sec on each side using a LED curing unit emitting 800 mW/cm^2^ of light intensity (Cure TC-01, Spring Health Products). After curing, the surface of the specimens was ground with a sequence of 320, 600 and 1200-grit SiC papers under running water. Finally, they were divided into four groups of fifty specimens each.

Ten polishers per type of polishing means (ONG, JS, OB, and JPB), forty in total, were used for polishing the two hundred resin composite specimens. Each cup/brush was used to polish five intact resin composite specimens, once before polisher sterilization and then after each of the four sterilization cycles. Polishing was performed by a single operator, maintaining a constant pressure for 20 s, using a slow-speed hand piece according to the manufacturer’s instructions. After their use, the cups and brushes were examined under an optical microscope at 1.6× magnification (M80, Leica), sonicated in an ultrasonic cleaning bath for 5 min (Biosonic uc 125, Coltene) and sterilized, using an autoclave at 134 °C for 20 min (2340E, Tuttnauer). Re-evaluation of the sterilized instruments under the optical microscope followed. Subsequently, they were used for processing new specimens.

After polishing, the resin composite specimens were rinsed out and dried with an air/water syringe for 30 s. Surface roughness parameters Sa (average roughness), Sz (ten point height over the complete 3D surface), Sdr (developed interfacial area ratio), and Sci (core fluid retention index) were evaluated using an optical profilometer (Wyko NT1100, Veeco) and surface gloss using a glossmeter at 60° (Novo Curve, Rhopoint instruments). Four measurements were recorded for gloss, following a 90° rotation of the specimens, and they were averaged to obtain the mean value for each specimen. The values recorded after the first application of the polishing instruments, prior to any sterilization cycle, were considered as those of a control group.

The results were analyzed using the non-parametric Friedman test to investigate differences among the related groups and the Wilcoxon signed-rank tests as post hoc analysis in order to determine which groups were statistically different. The level of significance was set at α = 0.05. The statistical software SPSS Statistics 22 was used for the analysis.

## 3. Results

**Figure 1:**
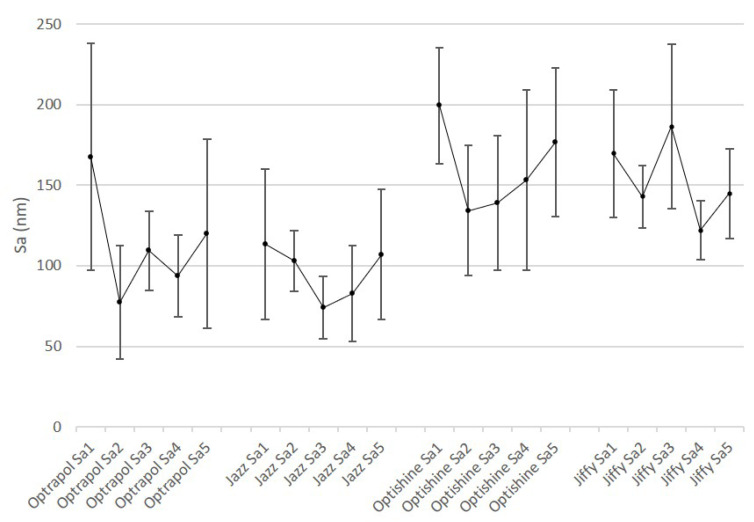
Sa mean values of all polishers at baseline and after each sterilization cycle (n = 10, *p* < 0.05).

**Figure 2:**
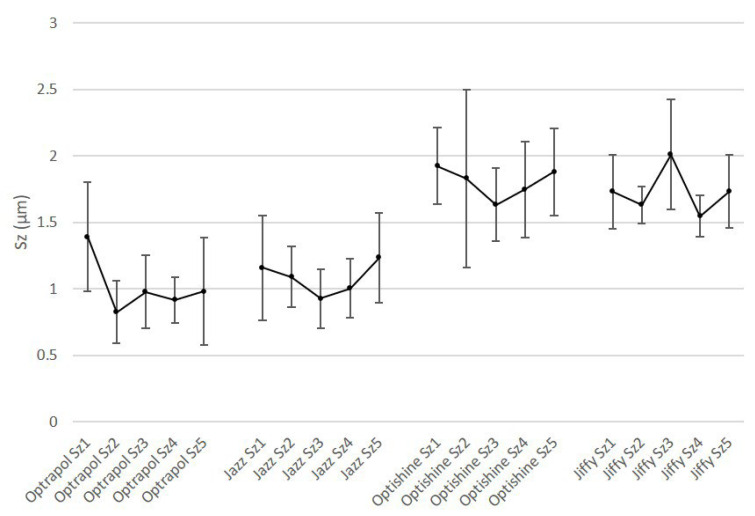
Sz mean values of all polishers at baseline and after each sterilization cycle (n = 10, *p* < 0.05).

**Figure 3:**
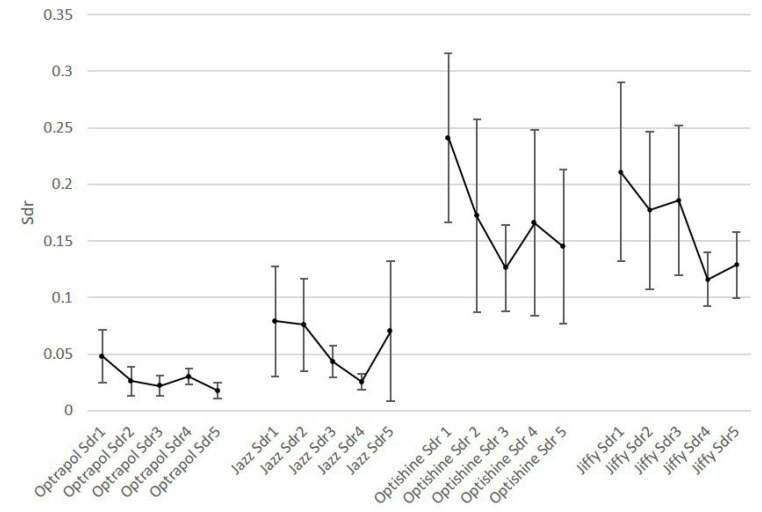
Sdr mean values of all polishers at baseline and after each sterilization cycle (n = 10, *p* < 0.05).

**Figure 4:**
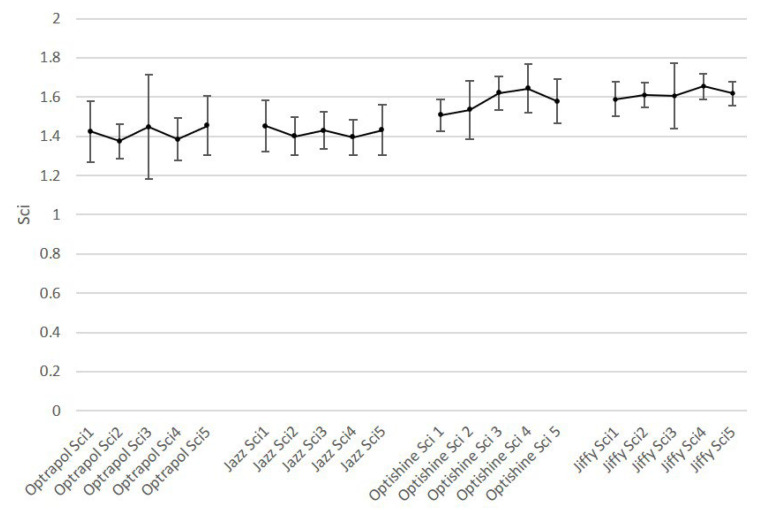
Sci mean values of all polishers at baseline and after each sterilization cycle (n = 10, *p* < 0.05).

**Figure 5:**
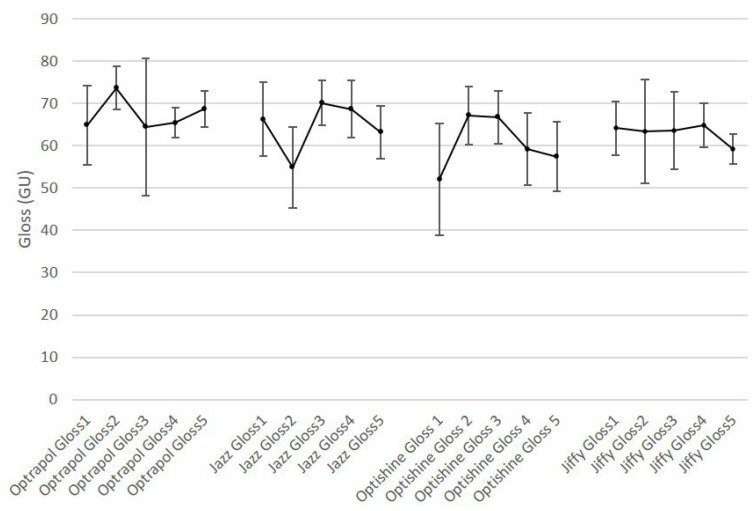
Gloss mean values of all polishers at baseline and after each sterilization cycle (n = 10, *p* < 0.05).

Figure 1, Figure 2, Figure 3, Figure 4 and Figure 5 present the roughness and gloss values of the nanohybrid resin composite, while Table 2 presents the differences between the values recorded after each sterilization cycle (2–5) in relation to the first application of the polishing tool (1).

### 3.1. Optrapol Next Generation

For ONG, all Sa and Sz differences presented negative values, which means that higher values were recorded after the first application for both roughness parameters. After the tool had been subjected to sterilization cycles, the differences became gradually smaller. However, this reduction was statistically significant only for ΔSa2_1 relative to ΔSa5_1.

The mean difference values for Sdr showed a statistically significant difference only between ΔSdr4_1 and ΔSdr5_1.

The mean difference values for Sci did not show any statistically significant difference.

Regarding gloss, the biggest difference value was recorded after the 1st sterilization cycle (ΔGloss2_1) while the following were lower. Gloss differences, though, were not statistically significant.

**Figure 6:**
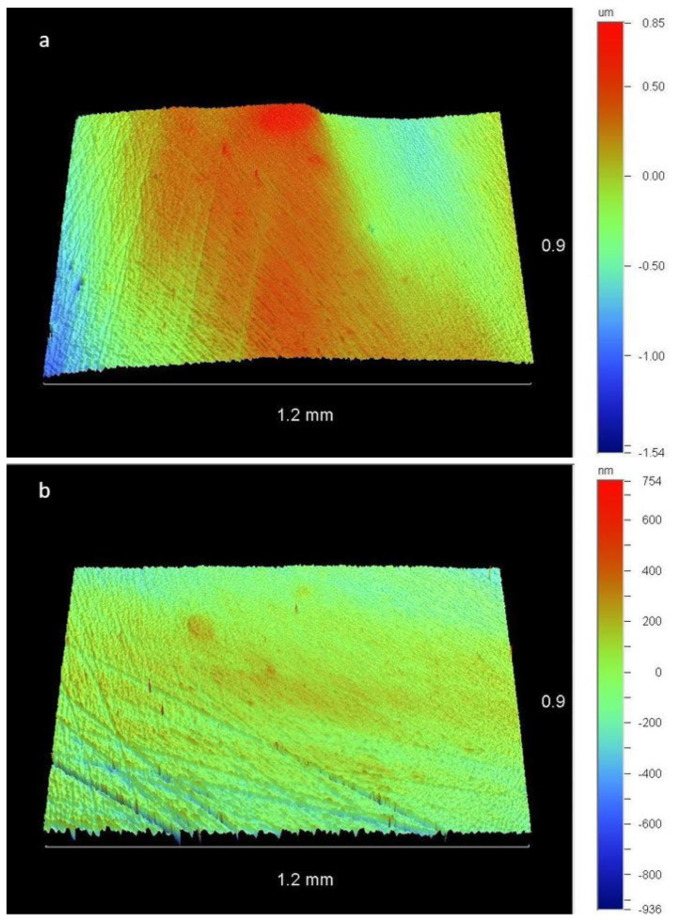
Resin composite surfaces after polishing with ONG. (**a**) Sample-3b (after the first sterilization cycle). (**b**) Sample-3e (after the fourth sterilization cycle). The green area corresponds to the smoothest surface, while the red area corresponds to increased roughness in the form of peaks.

Figure 6 shows representative images, captured by the optical profilometer, of a sample polished with ONG, after one and four sterilization cycles, allowing a qualitative assessment of the resin composite surface. 

**Figure 7:**
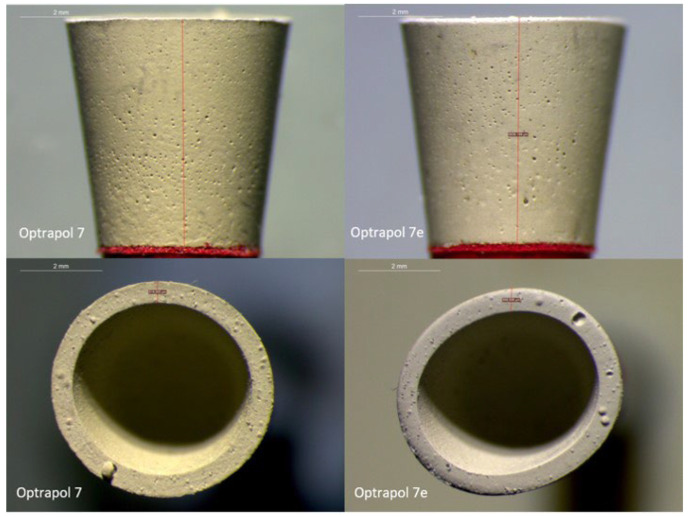
Representative optical microscope images (1.6×) of ONG before use (sample 7-left side) and after four sterilization cycles and five applications (sample 7e-right side).

The qualitative assessment of ONG’s surface and shape, by the optical microscope, revealed a gradual decrease in the porosity over the course of sterilizations. The sharp edges before the first application were rounded after sterilization cycles and following use. A shape change was also noticed from round to elliptical (Figure 7).

### 3.2. Jazz Supreme

For JS, the Sa biggest difference was observed between the first and the third application (ΔSa3_1), with Sa decreasing after the first sterilization. The differences ΔSa3_1, ΔSa4_1, and ΔSa5_1 revealed a reduction of Sa after the third application, denoting a trend for qualitative improvement of the resin composite surface, but not to a statistically significant level (*p* = 0.053).

ΔSz values did not show any statistically significant differences. Τhe biggest difference was noted between the first and the third application (ΔSz3_1), with Sz decreasing at the third application, yielding the smoothest surface.

Sdr was reduced in general. The biggest difference was that of ΔSdr4_1, where Sdr decreased to a statistically significant level, relative to ΔSdr2_1 (*p* = 0.022).

ΔSci values did not show any statistically significant differences. However, all differences had negative values, which means that after the first application, Sci had the biggest value, while the smallest occurred after the fourth application.

In terms of gloss, the biggest difference was recorded at the second application (ΔGloss2_1), where gloss decreased. At the next two applications, the differences ΔGloss3_1 and ΔGloss4_1 revealed a rise in gloss compared to the first application. This was statistically significant (*p* = 0.01 and *p* = 0.02, respectively) compared to ΔGloss2_1. Finally, after the fifth application, gloss appeared lower than after the first, while the difference ΔGloss5_1 differed statistically significantly from ΔGloss2_1 (*p* = 0.03) and ΔGloss3_1 (*p* = 0.02).

**Figure 8:**
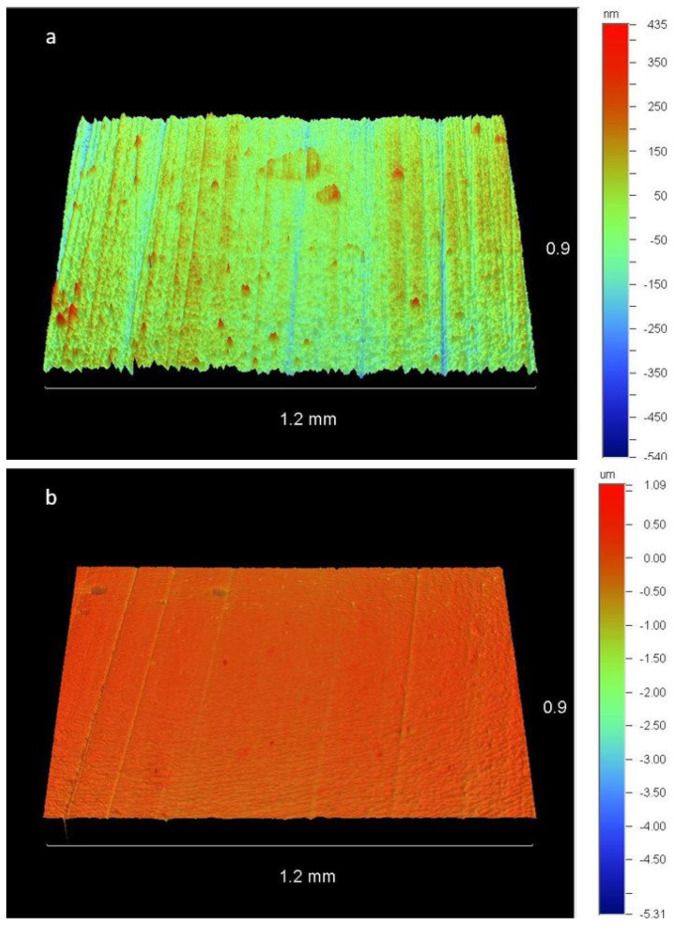
Resin composite surfaces after polishing with JS. (**a**) Sample-6d (after the third sterilization cycle). The green area (0.00) corresponds to the smoothest surface. (**b**) Sample-6e (after the fourth sterilization cycle). The red-brown area (−0.50) indicates valleys.

Figure 8 shows representative images captured by the optical profilometer of a sample polished with JS after three and four sterilization cycles, allowing a qualitative assessment of the resin composite surface. 

**Figure 9:**
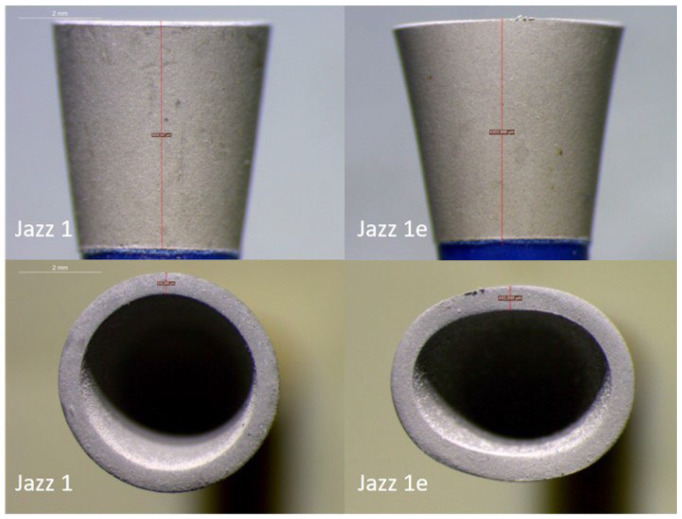
Representative optical microscope images (1.6×) of JS before use (sample 1-left side) and after four sterilization cycles and five applications (sample 1e-right side).

JS presented no initial porosity, which remained unchanged throughout the four sterilization cycles. There was a mild rounding of the edges and a mild change of its shape from round to elliptical as a result of sterilization (Figure 9). Debris accumulation was obvious after each application, which could be entirely removed after placement in the ultrasonic bath.

### 3.3. Optishine Brush

For OB, the mean difference values for Sa and Sz were negative, meaning that after the first application, Sa and Sz were higher than after all of the following applications. The biggest differences were observed for ΔSa2_1 and ΔSz3_1, but they were statistically non-significant. The mean difference values of Sdr did not show any statistically significant differences among them. The highest Sdr value was recorded after the first application. Sci had the lowest value after the first application. The biggest difference occurred after the fourth application. The smaller changes compared to the first application were noted after the second and fifth application. Statistical analysis did not show any statistically significant differences.

Gloss showed the lowest value after the first application. The differences ΔGloss2_1 and ΔGloss3_1 were the biggest without a statistically significant difference between them. ΔGloss2_1 was statistically different from ΔGloss5_1 (*p* = 0.03) while ΔGloss3_1 from both ΔGloss4_1 (*p* = 0.01) and ΔGloss5_1 (*p* = 0.02).

**Figure 10:**
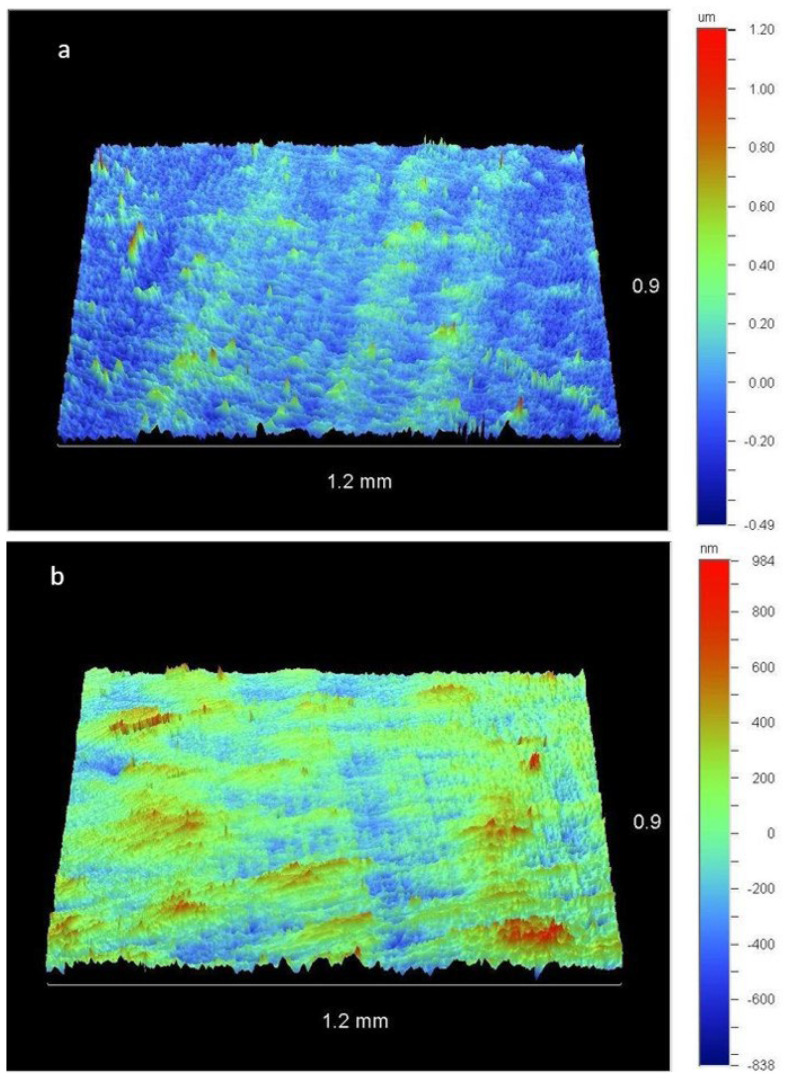
Resin composite surfaces after polishing with OB. (**a**) Sample-5b (after the first sterilization cycle). (**b**) Sample-5e (after the fourth sterilization cycle). A smoother surface was observed in the first case.

Figure 10 shows representative images captured by the optical profilometer of a sample polished with OB after one and four sterilization cycles, allowing a qualitative assessment of the resin composite surface. 

**Figure 11:**
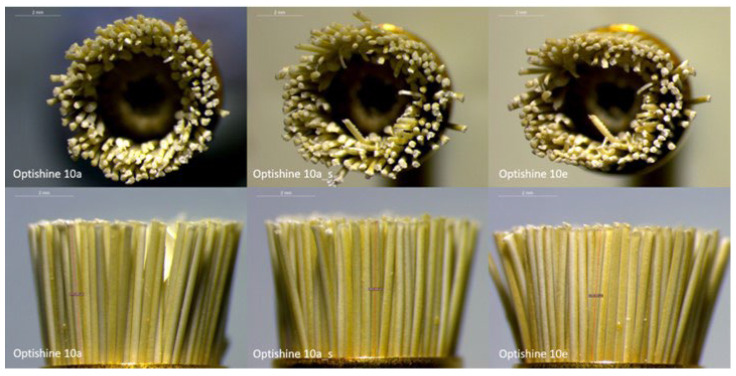
Representative optical microscope images (1.6×) of OB after the first application (10a), after the first application and sterilization (10 a_s), and after the fifth application (10e). A change of shape, a reduction of the tool’s diameter, and bending of some bristles was observed.

The qualitative assessment of OB by the optical microscope showed that even though the shape after the first application remained almost as its original, after sterilization there was a significant change. Some bristles were bent and deviated from their original direction and a kind of rip was also observed along the long axis (Figure 11).

### 3.4. Jiffy Polishing Brush

For JPB, the lowest Sa value was observed after the fourth application and the difference ΔSa4_1 was the greatest and statistically significantly greater than all others. At the same time, there was a statistically significant difference between ΔSa2_1 and ΔSa3_1 (*p* = 0.02). The third application was the only one that had Sa greater than the first application.

The biggest difference of Sz was ΔSz3_1, where the third application had a higher value than the first. The differences ΔSz2_1 and ΔSz4_1 were statistically significantly smaller than ΔSz3_1 (*p* = 0.01 in both cases). The negative value of the differences ΔSz2_1 and ΔSz4_1 indicated that the first application had bigger Sz than the second and the fourth.

Regarding Sdr, the first application presented the highest value of all the following. The biggest differences and therefore the smallest Sdr values were those of ΔSdr4_1 and ΔSdr5_1. The differences ΔSdr2_1 and ΔSdr3_1 were statistically significantly smaller than the previous two.

The mean values of Sci differences did not show any statistically significant differences. The highest value was observed after the fourth application.

Finally, the mean gloss differences did not show any statistically significant difference as well. The highest value occurred after the fourth application.

**Figure 12:**
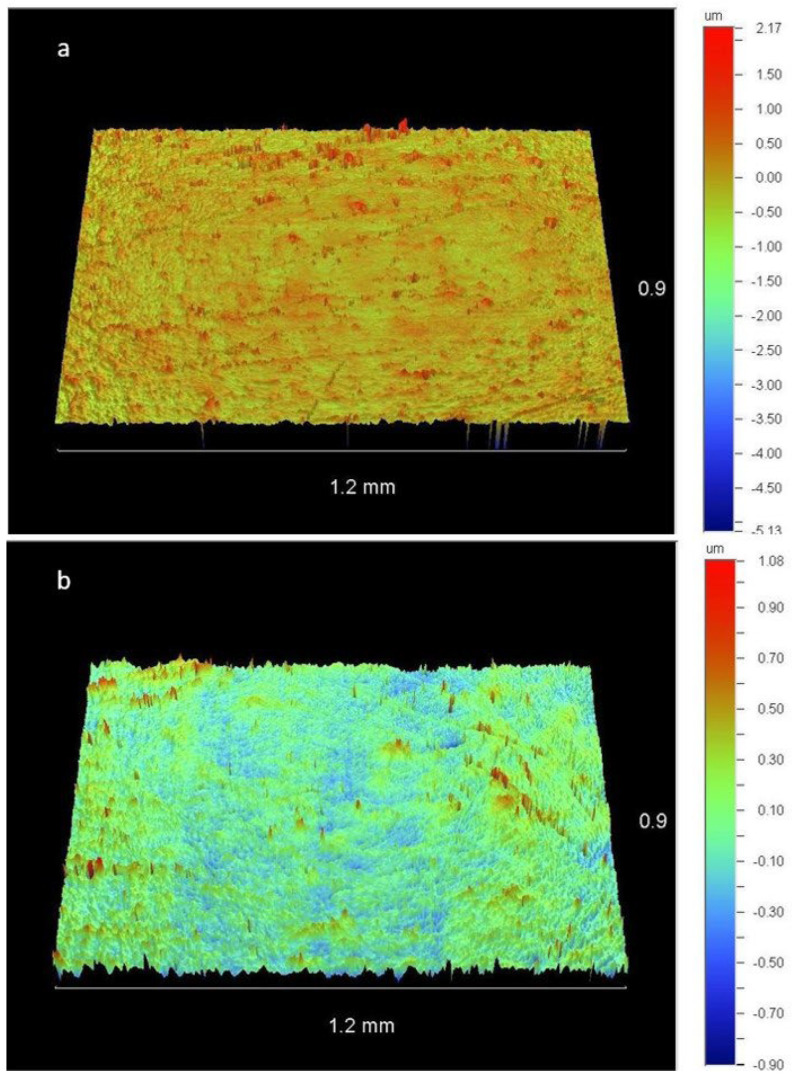
Resin composite surfaces after polishing with JPB. (**a**) Sample-5c (after the second sterilization cycle). (**b**) Sample-5d (after the third sterilization cycle). A smoother surface was observed in the second case.

Figure 12 shows representative images captured by the optical profilometer of a sample polished with JPB after two and three sterilization cycles, allowing a qualitative assessment of the resin composite surface. 

**Figure 13:**
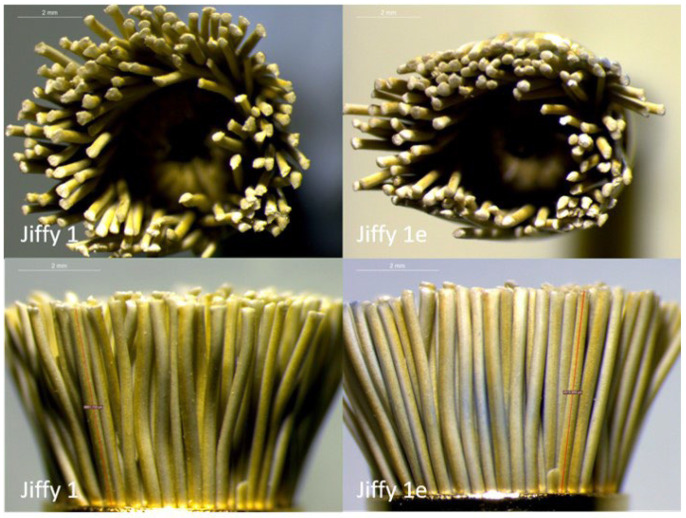
Representative optical microscope images (1.6×) of JPB after the first (1) and after the fifth application (1e). A gradual fusion of the bristles, a decrease in the tool’s diameter, and rounding of the bristle tops was observed.

The qualitative assessment of JPB by the optical microscope showed that the bristles, which were initially clearly separated, became gradually sintered with the progression of the sterilization cycles, resulting in a reduction of the tool’s diameter. Some bristles also became ripped along the long axis and their tops became rounded (Figure 13).

## 4. Discussion

The null hypothesis was rejected as all four polishing tools, ONG, JS, OB and JPB, presented a variation in their effectiveness of yielding a smooth surface.

Fillers of resin composites affect the surface quality of restorations. Their content, type, mean size, and distribution vary among different formulations of resin composites. Thus, only one nanohybrid resin composite was used to make all 200 samples in the present study to avoid any confounding factor.

At the same time, four S parameters, developed to address the three-dimensional nature of the surface texture, were examined to make surface characterization as complete as possible. Surface morphology was evaluated using two amplitude parameters (Sa, Sz), one hybrid (Sdr) and one functional (Sci) [33,34,35,36].

Sa (or Ra in other cases), the average roughness evaluated over the complete surface, is the most widely used parameter by researchers studying surface texture [3,20,24,37,38,39]. It represents an overall measure of the surface texture and is used to indicate significant deviations in the texture characteristics. However, Sa does not provide detailed information about the type and the shape of the profile, as it is insensitive to differentiating peaks from valleys and the spacing of the various texture features. In contrast, the Sz parameter, which represents the average difference between the five highest peaks and five lowest valleys, is more representative of surface roughness because it cannot be as affected by outliers, and because it reveals differences when comparing two surfaces more clearly and sooner than Sa. It is useful particularly when studying wear mechanisms. 

The hybrid parameter Sdr is expressed as the percentage of the additional surface area contributed by the texture as compared to an ideal plane. It is associated with the slopes of the peaks and may further differentiate surfaces of similar amplitudes and average roughness. Sdr increases with the spatial intricacy of the texture whether or not Sa changes. It is useful in applications involving adhesion. Sdr may also find applications related to the manner in which light is scattered from a surface. 

Finally, the functional parameter Sci is used to compare the tribological properties of surfaces with different average roughness. Sci relates to the extent of fluid retention on a surface, and therefore possesses a central position among the parameters when testing biomaterials [40]. In the present study, there was no group without undergoing sterilization, as such a group would not correspond to the clinical reality. The tools were not disinfected in order to avoid any chemical agent involvement in a possible degradation process. Disinfected tools may be affected by factors such as the selected solution, its concentration, and the time they remained in it, and this would lead to faster wear [41]. This has been attributed to the degradation of the softer rubber substrate, which gets abraded more easily during the application of the tool.

Finishing of the resin composite samples with a fine diamond or a multi-fluted bur better resembles the clinical practice, and, thus, several studies use such tools as part of their methodology prior to polishing [3,19,42,43,44]. However, the surface that those tools leave shows heterogeneity in the degree of grinding and, therefore, heterogeneity in surface roughness. Thus, a metallographic grinding device has been used in many other studies for finishing, including the present one [21,22,23,24,37,38,41,45,46,47,48,49,50]. The abrasiveness of the papers used corresponded to the abrasiveness of fine diamonds.

The pressure exerted when applying finishing polishing tools appears to affect both surface roughness and surface gloss [41]. However, there were few studies indicating the application force, which was in the order of 1–2 N [41,48,51]. Heintze et al. [51] observed that a 2 N force provided smoother surfaces, with greater gloss, than a 4 N force. It was concluded that the subjective estimation of the applied force during grinding could be the cause of different results among studies using the same materials (resin composite and finishing/polishing system), making the result comparison difficult. Often, in the methodology of various studies, it is indicated that finishing and polishing was carried out by the same operator with constant pressure on all samples without accurately measuring the force exerted [3,19,22,23,24,37,38,42,43,44,45,46,47,49,50]. This was also applied in the current study because it is more relevant to clinical practice. A single calibrated operator polished all samples, applying approximately a 2 N force.

Another factor affecting the surface quality of resin composites is the time spent for polishing. The application time of the polishing tools varies usually between 20 s and 40 s [21,23,24,42,44]. It was found that increasing the time from 5 s to 30 s, the quality of the surface was improved to a statistically significant degree [51]. In addition, the rotation speed of the tools varied, as well as spraying the water or not. In the present study, as in most of the literature, the rotation speed and the water spraying were both in accordance with the manufacturers’ instructions in order to achieve the optimum performance of the tools.

ONG, JS, and OB yielded the coarsest surfaces (highest Sa) on resin composites after the first application, i.e., before the first sterilization cycle. One possible explanation is associated with the manufacturing process of abrasives, which may leave a rigid surface of the rubber cup or brush bristles. After the first application, the surface of the tool may get softened due to friction and heat development with the resin composite surface. At the same time, sterilization may soften the tool through the raised temperature and the impact of steam and make it less aggressive in cutting. Therefore, the tool becomes capable of yielding a smoother surface. This interpretation agrees with the appearance of the two different rubber cups used, as it seems that their sharp edges were blunted even after the first sterilization cycle.

ONG presented its best performance at the second application, i.e., after the first sterilization cycle. This concerns the parameters Sa, Sz, Sci, and gloss with the exemption of Sdr, whose smallest value occurred at the third application, i.e., after the second sterilization cycle. However, it was not statistically significantly different from the other applications.

The surface quality polished with ONG showed a tendency to degrade at the fifth application, i.e., after the fourth sterilization cycle, as Sa, Sz, and Sci increased. However, the degradation was statistically significant only for Sa. Regarding the other parameters (Sz, Sci, gloss), there was no statistically significant changes with the progress of the sterilization cycles. This degradation could be explained by the porosity increase in the rubber cup observed and possibly due to fatigue of the silicone substrate in which the diamond particles were embedded. The loss of diamond particles as a result of use may also have contributed, leading to a reduction of the polisher’s abrasive capability.

JS at the first two applications yielded resin composite surfaces with higher surface roughness and lower gloss. After the third and fourth applications, there was a statistically significant improvement of gloss, while the lowest Sdr value was noted. Sz showed a temporary improvement after the second application while Sci remained almost unchanged.

At the last application of JS, the values of all parameters tended to deteriorate, but only gloss deteriorated to a statistically significant degree. The reduction of the tool’s effectiveness could be attributed to the same features of fatigue mentioned already for ONG. In addition, for JS, the debris that appeared to remain on the tool surface was likely to cover the interspaces between the abrasive particles, thus limiting its abrasive capability.

OB did not show statistically significant changes of the parameters Sa and Sz. However, a tendency of reduced efficiency could be observed after the third sterilization cycle. The observations on Sdr were similar.

Sci of resin composite surfaces polished with OB tended to increase along with the progression of sterilization cycles, but not to a statistically significant level. 

In terms of gloss, there was a statistically significant improvement after the first two sterilization cycles. Afterwards, there was a deterioration in the performance of the tool, which was also proven to be statistically significant. The gradual deformation of the brush bristles, which carry silicon carbide as the abrasive agent, was the most possible cause of the degradation of the abrasive capability of OB.

The lack of a specific pattern between sterilization cycles regarding the tool’s ability to render a resin composite surface smooth is noteworthy for JPB. With the exception of Sci and gloss, which did not show statistically significant changes after four sterilization cycles, the other three parameters (Sa, Sz, Sdr) presented fluctuating, statistically significant changes among the applications. After the second sterilization cycle, the tool yielded the worst resin composite surface, and after the third sterilization cycle, it yielded the best. Subsequently, i.e., after the fourth sterilization cycle, the effectiveness of the tool was re-degraded to a statistically significant extent. This was difficult to interpret, making the tool’s behavior after sterilization cycles and repetitive uses unpredictable. The fusion of the bristles observed was probably due to the high temperature of sterilization in parallel with other fatigue characteristics, which led to randomly deformed tools, resulting in a non-consistent polishing effect.

There are very few studies in the literature that have dealt with the effect of sterilization on the effectiveness of resin composite polishing means [37,41]. The difficulty in comparing the results of the current study with those of the aforementioned studies was that different polishing systems and resin composites were used as well as different methodologies for determining surface roughness. Therefore, no safe conclusions can be drawn with regard to the agreement or not of the results.

Tate et al. [37] used two hybrid resin composites and two one-stage polishing systems. Those tools were divided into three groups. In the first group, they were re-used for three applications, without being subjected to any sterilization method. In the second group, they were sterilized in an autoclave, and in the third group, in a microwave furnace. It was noteworthy that after three applications on a hybrid resin composite, the tools that had not undergone any treatment yielded rougher resin composite surfaces than those that had been sterilized three times in either way. This underlines the importance of wear during application, even without disinfection or sterilization. The researchers attributed this result to the loss of the abrasive grains, which reduced the polishing capability.

In contrast, tools sterilized in an autoclave or a microwave furnace showed an increased connection of the abrasive grains to the matrix, which was attributed to the development of pressure and heat, respectively. When tools were sterilized in an autoclave, the surface roughness increased after three sterilization cycles, but not to a statistically significant degree. It was concluded that the tools studied could be sterilized at least three times without their effectiveness being reduced. This result seems to be consistent with the results of the present study, where the quality of resin surfaces yielded by the polishing tools was generally and progressively degraded. However, the comparison of the results must be conducted with caution, since except for the materials, the roughness parameters were assessed differed.

In the study of Heinze and Forjanic [41], the tools were divided into three groups. In the first group, the tools were not treated; in the second, they were sterilized in an autoclave; and in the third, they were disinfected before they had been sterilized in an autoclave. In this study too, the greatest decrease in the performance of the tool happened to the untreated group, both for surface roughness and gloss. This finding reconfirmed that wear during use, even in the absence of sterilization or disinfection, can be the cause of gradually inferior performance. When the tools were only sterilized, there was again a reduction in their effectiveness, but it was statistically significant only for gloss and not for surface roughness. This reduction was attributed to the fact that when rubbing the tool on the resin composite surface, it became softer relative to its state before the first application. This finding was also consistent with that of Tate et al. [37], as in both studies, Ra was not affected by sterilization to a significant extent. However, the findings of the current study are in part the opposite, as ONG and JPB presented increased Sa values to a statistically significant extent after the fourth sterilization cycle. On the other hand, JS and OB showed a tendency to have a reduced performance after four sterilization cycles regarding Sa and Sz, although this was not statistically significant.

Finally, when disinfection preceded sterilization, Heintze and Forjanic [41] observed an improvement in the effectiveness of the tools to a statistically significant degree for gloss and only a tendency of improvement for Ra. Disinfection possibly caused degradation and loss of part of the elastic substrate of the tool and exposure of the abrasive particles, resulting in improved abrasive capability but also faster wear.

In the present study, all Sa values for all tools ranged from 0.07 μm to 0.2 μm. Therefore, all resin composite surfaces could be considered smooth and clinically satisfactory. A possible disadvantage could be the relatively big standard deviations, which were attributed to the fact that no filter was used in the optical profilometer. The main function of filters is to isolate outliers, i.e., frequency bands that are not related to roughness, but rather to waviness and form errors [52]. The choice of the appropriate filter each time is to some extent arbitrary, so there is a risk of excluding values related to roughness. In the present investigation, in order to avoid excluding useful information about roughness, it was chosen not to use any filter and to take into account all measured values.

The findings of the present study showed a declination trend of the tools’ polishing ability after four sterilization cycles and five uses. However, the threshold of clinical acceptance was not surpassed. It might be interesting in a future work, in order to set a proposed number of uses, to further increase the number of sterilization cycles and uses until this limit is reached, always considering the wear and fatigue that may render the tools useless. 

## 5. Conclusions

Based on the results of the present study, the following conclusions can be drawn:

1. All polishers showed an improved polishing capability after the first sterilization cycle/second use compared to the first use.

2. ONG, JS, and OB, despite showing a trend of gradual declination in their polishing capability with the progression of sterilization cycles and uses, as reflected in almost all of the evaluated parameters, was not statistically significant.

3. JPB did not follow a specific polishing pattern in relation to the times of use and sterilization cycles, but its performance also declined after the fourth sterilization cycle.

4. The two polishing rubbers performed better than the two polishing brushes.

5. Although the two rubber abrasives exhibited a similar pattern of polishing behavior, the two polishing brushes did not follow a similar pattern.

6. The two abrasive rubbers showed mild, similar changes in relation to their original characteristics with the progression of sterilization cycles and times of use.

7. The wear of polishing brushes was significant, with JPB’s being more intense.

8. Even after several sterilization cycles, polishers retain a satisfactory polishing capability.

## Figures and Tables

**Table 1:** Single-step polishers and resin composite tested.

Polisher	Type	Shape	Manufacturer
Optrapol Next Generation Rubber Polisher (ONG)	Silicone polisher highly filled with micro-fine diamond crystallites (up to 72 wt.%)	Cup	Ivoclar Vivadent
Jazz Supreme Rubber Polisher (JS)	Synthetic rubber matrix infused with diamond particles in various sizes and pigments (mainly titanium dioxide)	Cup	SS White Dental
Optishine Brush (OB)	Silicon carbide polishing particles embedded in the bristles	Cup	Kerr
Jiffy Polishing Brush (JPB)	Silicon carbide polishing particles embedded in the bristles	Cup	Ultradent

**Resin composite**	**Composition**		**Manufacturer**
IPS Empress Direct Shade: Enamel A1	78.1% Ba-Al-fluorosilicate glass (Barium glass fillers 0.4–0.7 μm, Prepolymers 1–10 μm, Spherical mixed oxides 150 nm, Ytterbium trifluoride 100 nm) 21.5% Bis-GMA, UDMA, Tricyclodocane dimethanol dimethacrylate 0.4% Catalysts and stabilizers <0.1% Pigments		Ivoclar Vivadent

**Table 2:** Differences of all properties after each sterilization cycle. The statistical analysis refers only to the same property of a single polisher, not across polishers. Different superscripts at each column, per property, denote statistically significant differences. (n = 10, *p* < 0.05).

Cycle Numbers	Optrapol Next Generation	Jazz Supreme	Optishine Brush	Jiffy Polishing Brush
**ΔSa (mean ± SD) (nm)**				
2_1	−90.23 ^1^ ± 73.70	−10.34 ± 22.64	−66.22 ± 38.75	−26.92 ^1^ ± 34.40
3_1	−58.35 ^1,2^ ± 64.56	−39.43 ± 50.93	−60.55 ± 42.72	16.69 ^2^ ± 59.49
4_1	−73.84 ^1,2^ ± 81.48	−30.74 ± 48.17	−46.14 ± 62.05	−47.76 ^3^ ± 45.91
5_1	−47.94 ^2^ ± 115.09	−6.49 ± 52.63	−22.66 ± 48.62	−24.89 ^1,2^ ± 26.98
**ΔSz (mean ± SD) (μm)**				
2_1	−0.56 ± 0.47	−0.00 ± 0.25	−0.09 ± 0.64	−0.09 ^a,b^ ± 0.20
3_1	−0.41 ± 0.52	−0.23 ± 0.45	−0.29 ± 0.34	0.27 ^c^ ± 0.43
4_1	−0.47 ± 0.46	−0.16 ± 0.42	−0.18 ± 0.51	−0.18 ^a^ ± 0.22
5_1	−0.41 ± 0.73	0.08 ± 0.58	−0.04 ± 0.37	0.01 ^b,c^ ± 0.22
**ΔSdr (mean ± SD)**				
2_1	−0.02 ^A,B^ ± 0.02	−0.00 ^A^ ± 0.04	−0.07 ± 0.12	−0.03 ^A^ ± 0.05
3_1	−0.03 ^A,B^ ± 0.03	−0.03 ^A,B^ ± 0.06	−0.12 ± 0.07	−0.02 ^A^ ± 0.09
4_1	−0.02 ^A^ ± 0.02	−0.04 ^B^ ± 0.06	−0.08 ± 0.10	−0.09 ^B^ ± 0.07
5_1	−0.03 ^B^ ± 0.03	−0.01 ^A,B^ ± 0.08	−0.10 ± 0.10	−0.08 ^B^ ± 0.06
**ΔSci (mean ± SD)**				
2_1	−0.05 ± 0.17	−0.05 ± 0.13	0.03 ± 0.14	0.02 ± 0.14
3_1	0.02 ± 0.37	−0.02 ± 0.14	0.11 ± 0.13	0.01 ± 0.20
4_1	−0.04 ± 0.18	−0.06 ± 0.12	0.14 ± 0.12	0.06 ± 0.13
5_1	0.03 ± 0.25	−0.02 ± 0.11	0.07 ± 0.09	0.02 ± 0.09
**ΔGloss (mean ± SD) (GU)**				
2_1	8.83 ± 9.38	−11.38 ^i^ ± 10.75	15.10 ^i,ii^ ± 17.43	−0.68 ± 12.85
3_1	−0.36 ± 20.36	3.85 ^ii^ ± 10.33	14.72 ^i^ ± 13.31	−0.50 ± 12.25
4_1	0.57 ± 10.74	2.40 ^ii,iii^ ± 10.98	7.16 ^ii,iii^ ± 12.22	0.71 ± 8.18
5_1	3.84 ± 9.65	−3.06 ^iii^ ± 11.88	5.39 ^iii^ ± 13.61	−4.88 ± 6.76

## Data Availability

Data is contained within the article.
